# Caffeic acid phenethyl ester inhibits neuro-inflammation and oxidative stress following spinal cord injury by mitigating mitochondrial dysfunction via the SIRT1/PGC1α/DRP1 signaling pathway

**DOI:** 10.1186/s12967-024-05089-8

**Published:** 2024-03-25

**Authors:** Yanan Zhang, Qian Deng, Hongxiang Hong, Zhanyang Qian, Bowen Wan, Mingjie Xia

**Affiliations:** 1 Department of Orthopedics, Taizhou People’s Hospital of Nanjing Medical University, Taizhou School of Clinical Medicine, Nanjing Medical University, Taizhou, China; 2 Department of Spine Surgery, Nantong First People’s Hospital, The Second Affiliated Hospital of Nantong University, Research Institute for Spine and Spinal Cord Disease of Nantong University, No. 666, ShengLi Road, Chongchuan District, Nantong, Jiangsu China; 3 Postgraduate School, Nanjing University of Chinese Medicine, Nanjing, China; 4 Department of Orthopedics, Northern Jiangsu People’s Hospital Affiliated to Yangzhou University/Clinical Medical College, Yangzhou University, Yangzhou, China

**Keywords:** Spinal cord injury, CAPE, Neuro-inflammation, Oxidative stress, SIRT1

## Abstract

**Background:**

The treatment of spinal cord injury (SCI) has always been a significant research focus of clinical neuroscience, with inhibition of microglia-mediated neuro-inflammation as well as oxidative stress key to successful SCI patient treatment. Caffeic acid phenethyl ester (CAPE), a compound extracted from propolis, has both anti-inflammatory and anti-oxidative effects, but its SCI therapeutic effects have rarely been reported.

**Methods:**

We constructed a mouse spinal cord contusion model and administered CAPE intraperitoneally for 7 consecutive days after injury, and methylprednisolone (MP) was used as a positive control. Hematoxylin–eosin, Nissl, and Luxol Fast Blue staining were used to assess the effect of CAPE on the structures of nervous tissue after SCI. Basso Mouse Scale scores and footprint analysis were used to explore the effect of CAPE on the recovery of motor function by SCI mice. Western blot analysis and immunofluorescence staining assessed levels of inflammatory mediators and oxidative stress-related proteins both in vivo and in vitro after CAPE treatment. Further, reactive oxygen species (ROS) within the cytoplasm were detected using an ROS kit. Changes in mitochondrial membrane potential after CAPE treatment were detected with 5,5′,6,6′-tetrachloro-1,1′,3,3′-tetraethyl-imidacarbocyanine iodide. Mechanistically, western blot analysis and immunofluorescence staining were used to examine the effect of CAPE on the SIRT1/PGC1α/DRP1 signaling pathway.

**Results:**

CAPE-treated SCI mice showed less neuronal tissue loss, more neuronal survival, and reduced demyelination. Interestingly, SCI mice treated with CAPE showed better recovery of motor function. CAPE treatment reduced the expression of inflammatory and oxidative mediators, including iNOS, COX-2, TNF-α, IL-1β, 1L-6, NOX-2, and NOX-4, as well as the positive control MP both in vitro and in vivo. In addition, molecular docking experiments showed that CAPE had a high affinity for SIRT1, and that CAPE treatment significantly activated SIRT1 and PGC1α, with down-regulation of DRP1. Further, CAPE treatment significantly reduced the level of ROS in cellular cytoplasm and increased the mitochondrial membrane potential, which improved normal mitochondrial function. After administering the SIRT1 inhibitor nicotinamide, the effect of CAPE on neuro-inflammation and oxidative stress was reversed.On the contrary, SIRT1 agonist SRT2183 further enhanced the anti-inflammatory and antioxidant effects of CAPE, indicating that the anti-inflammatory and anti-oxidative stress effects of CAPE after SCI were dependent on SIRT1.

**Conclusion:**

CAPE inhibits microglia-mediated neuro-inflammation and oxidative stress and supports mitochondrial function by regulating the SIRT1/PGC1α/DRP1 signaling pathway after SCI. These effects demonstrate that CAPE reduces nerve tissue damage. Therefore, CAPE is a potential drug for the treatment of SCI through production of anti-inflammatory and anti-oxidative stress effects.

**Graphical Abstract:**

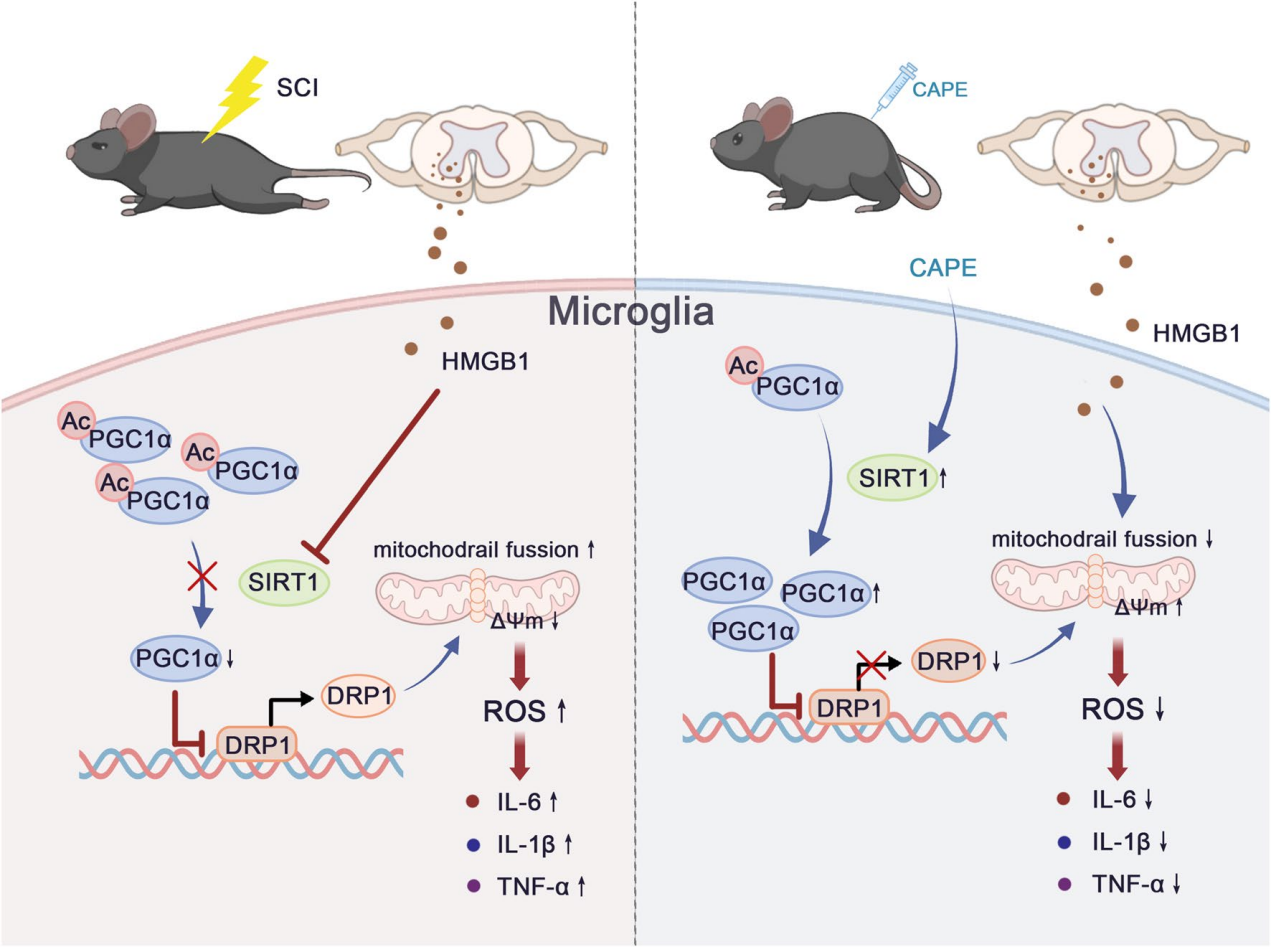

**Supplementary Information:**

The online version contains supplementary material available at 10.1186/s12967-024-05089-8.

## Introduction

Spinal cord injury (SCI) often results in severe and irreversible disability, due to sensory and motor dysfunction [1–3]. The SCI pathological process includes primary injury, which is defined as mechanical injury immediately occurring at the site of injury, and secondary injury, including pathological phenomena such as bleeding, edema, neuro-inflammation, and oxidative stress [4, 5]. Uncontrollable secondary injury can further aggravate nervous tissue destruction, which produces poorer outcomes. Inhibiting secondary injuries due to neuro-inflammation and oxidative stress is essential to better outcomes, with early intervention (inhibition) considered “the golden window” for SCI treatment [6–8].

Microglia-mediated neuro-inflammation after SCI is the main cause of nervous tissue damage [9]. Microglia are resident immune cells of the nervous system that play a positive role in physiological protection of normal nerve function [10]. However, microglia are activated by external stimulation after SCI and release a large number of inflammatory factors and mediators including tumor necrosis factor-alpha (TNF-α), interleukin-1 beta (IL-1β), 1L-6, cyclooxygenase-2 (COX-2), and inducible nitric oxide synthase (iNOS) [11–13]. The release of abundant inflammatory components can cause an inflammatory cascade that further aggravates the production of excessive intracellular reactive oxygen species (ROS) [14]. ROS-induced oxidative stress leads to the destruction of intracellular mitochondria, further disrupting the biological function of nerve cells [15, 16]. Therefore, inhibiting the damage caused by microglia-mediated neuro-inflammation and oxidative stress is essential to improve cell function and offer better outcomes for patients with SCI.

Recently, anti-neuroinflammatory drugs have been shown to produce therapeutic effects that improve motor functional recovery in SCI animal models [17]. Caffeic acid phenethyl ester (CAPE) is a main component of propolis. Propolis has anti-inflammatory, anti-oxidant, and anti-cancer activities and these activities are thought to contribute to the beneficial effects of propolis [18–20]. CAPE has been shown to reduce neurodegenerative disease progression [21]. Studies have shown that CAPE inhibits amyloid β-peptide-induced neuro-inflammation and oxidative stress through modulation of the Nrf2/HO-1 pathway in Alzheimer’s disease patients [22]. Further, CAPE treatment promotes microglial M2 polarization and suppresses oxidative stress, producing a neuroprotective effect for postoperative cognitive dysfunction patients [23]. Although previous authors demonstrated CAPE to reduce inflammatory mediators after SCI [24], the regulatory effects and specific mechanisms of CAPE action on microglia-mediated neuro-inflammation and oxidative stress are unknown.

Here, we demonstrate that CAPE effectively inhibits microglia-mediated neuro-inflammation and oxidative damage by activating the SIRT-1/PGC1-α signaling pathway. This reduces the destruction of cellular mitochondria by inhibiting DRP1. Furthermore, we found CAPE treatment significantly mitigates the destruction of nervous tissue after SCI and promotes the recovery of motor function in SCI mice. These results suggest that CAPE is neuroprotective and can be used as a therapeutic agent after SCI.

## Methods

### Animals

Sixty mice, each weighing approximately 25 g and aged 6–8 weeks were provided by Nanjing Medical University. The mice were raised in a specific pathogen-free laboratory center at Nanjing Medical University in standard cages (five mice per cage), with a 12 h/12 h light/dark cycle. The mice were provided with plenty of food and water in a comfortable environment with temperatures between 22 °C and 25 °C.

### Cell culture

BV-2 microglial cells were purchased from Procell Life Science & Technology Co., Ltd. (Wuhan, China) and cultured in Dulbecco’s modified Eagle medium (DMEM; KeyGEN, Nanjing, China) containing 10% fetal bovine serum (FBS; Gibco, Grand Island, NY, USA). To BV-2 microglia was added 10, 20 and 50 μM CAPE (HY-N0274, MedChemExpresss, Weehawken, USA) for 3 h, after which 100 ng/mL of HMGB1 (Sigma-Aldrich, St. Louis, Missouri, USA) was added for 12 h to induce neuro-inflammation and oxidative stress. Methylprednisolone (MP, 10 µg/ml) was used as a positive control.

### SCI model and CAPE treatment

The SCI surgical procedure was performed as in our previous study [25]. Briefly, mice were anesthetized with ketamine (80 mg/kg) by intraperitoneal injection. An impactor (RWD, Shenzhen, China) was used to strike the spinal cord (5 g × 5 cm impact force) on the T10 vertebrae, which produced a contusion. Tail-Flick reflex and hind limb paralysis appeared after injury indicating successful modeling. We randomly divided the mice into six groups: a. the Sham group, the mice only received laminectomy without damaging the spinal cord; b. the SCI group, the mice underwent surgery for SCI; c, d and e. the SCI + CAPE groups, the mice were treated with 10, 20 and 40 mg/kg of CAPE by daily intraperitoneal injection for 1 week after injury. f. the SCI + MP group, the mice were treated with 30 mg/kg of MP which used as a positive control. In this manner, the therapeutic effect of CAPE in SCI mice was evaluated.

### Western blot (WB) analysis

Proteins from BV-2 microglial cells and spinal cord tissues were extracted and quantified to ensure equal loading. These proteins were separated by size by sodium dodecyl sulfate–polyacrylamide gel electrophoresis (SDS-PAGE), followed by transfer to polyvinylidene difluoride (PVDF) membranes. After blocking to reduce background, membranes were incubated with primary antibodies targeting proteins. Subsequent washing steps were performed to remove unbound primary antibodies and the membranes were incubated with secondary antibody. Band density was assessed by chemiluminescence and analyzed with ImageJ software (National Institutes of Health, Bethesda, MD, USA). Regarding the quantification of WB bands, the brief method is as follows: import the Western blot images into ImageJ and complete background inversion. Use the selection tools in ImageJ to define regions of interest (ROIs) corresponding to individual lanes and protein bands on the Western blot images. Once ROIs are defined, use ImageJ’s measurement tools to quantify the intensity of protein bands within the selected regions. This is typically done by measuring the integrated density or mean intensity of each band. Normalize the intensity of protein bands to an appropriate loading control or reference protein to account for variations in protein loading or transfer efficiency. Details of the primary and secondary antibodies are shown in Table 1.

**Table 1:** Antibodies information

Antibodies name #Cat. No	Source	Species	Application	Dilution rate
Anti-iNOS antibody #ab15323	Abcam	Rb	WB, IF	1:250, 1:100
NOX2 Polyclonal antibody #19,013–1-AP	Proteintech	Rb	WB, IF	1:1000, 1:200
NADPH oxidase 4/NOX4 Rabbit mAb #48,782	SAB	Rb	WB, IF	1:1000, 1:200
COX2 (D5H5) XP^®^ Rabbit mAb #12,282	CST	Rb	WB, IF	1:1000, 1:200
SIRT1 Polyclonal antibody #13,161–1-AP	Proteintech	Rb	WB, IF	1:1000, 1:200
PGC1a Monoclonal antibody #66,369–1-Ig	Proteintech	Rb	WB	1:1000
DRP1 (C-terminal) Polyclonal antibody, #12,957–1-AP	Proteintech	Rb	WB, IF	1:1000, 1:100
TOM20 Polyclonal antibody, #11,802–1-AP	Proteintech	Rb	IF	1:100
HRP-conjugated Beta Actin Monoclonal antibody #HRP-60008	Proteintech	Ms	WB	1:10,000
Goat Anti-Rabbit IgG Secondary antibody (H + L), HRP #YFSA02	YIFEIXUE BioTech	Goat	WB	1:10,000
Goat Anti-Mouse IgG Secondary antibody (H + L), HRP # YFSA01	YIFEIXUE BioTech	Goat	WB	1:10,000
Anti-Iba1 antibody [EPR16588] #ab178846	Abcam	Rb	IF	1:500
Alexa Fluor^®^ 594 AffiniPure Fab Fragment Goat Anti-Rabbit IgG (H + L) #111,587,003	Jackson ImmunoResearch	Goat	IF	1:500
Alexa Fluor^®^ 488 AffiniPure Fab Fragment Goat Anti-Rabbit IgG (H + L) #111,547,003	Jackson ImmunoResearch	Goat	IF	1:500
Alexa Fluor^®^ 594 AffiniPure F(ab’)_2_ Fragment Goat Anti-Mouse IgG (H + L) #115,586,003	Jackson ImmunoResearch	Goat	IF	1:500
Alexa Fluor^®^ 488 AffiniPure F(ab’)_2_ Fragment Goat Anti-Mouse IgG (H + L) #115546,003	Jackson ImmunoResearch	Goat	IF	1:500

®: the registered trademark; #: the antibody number

### Real-time quantitative polymerase chain reaction (qPCR)

BV-2 microglial cells and spinal cord tissues were extracted for RNA using VeZol Reagent (R411-01, Vazyme, Nanjing, China). HiScript II 1st Strand cDNA Synthesis Kits (R211-02, Vazyme) were used for reverse transcription and processed with the ChamQ SYBR qPCR Master Mix (High ROX Premixed, Q341-02, Vazyme), which includes primers, cDNA, nucleases, buffer, and DNA polymerase. The qPCR reaction was completed with a Roche LightCycler 480 (Roche, Basel, Switzerland). For data analysis, a standard curve was used to convert Ct values into relative target concentrations. Details of the primer sequences are listed in Table 2.

**Table 2:** Primer sequences information

Gene name	Forward Sequence (5′-3′)	Reverse Sequence (5′-3′)
GAPDH	AGGTCGGTGTGAACGGATTTG	GGGGTCGTTGATGGCAACA
IL-1beta	GAAATGCCACCTTTTGACAGTG	TGGATGCTCTCATCAGGACAG
IL-6	CTGCAAGAGACTTCCATCCAG	AGTGGTATAGACAGGTCTGTTGG
iNOS	GTTCTCAGCCCAACAATACAAGA	GTGGACGGGTCGATGTCAC
TNF-alpha	CAGGCGGTGCCTATGTCTC	CGATCACCCCGAAGTTCAGTAG
NOX-4	TGCCTGCTCATTTGGCTGT	CCGGCACATAGGTAAAAGGATG
NOX-2	AGTGCGTGTTGCTCGACAA	GCGGTGTGCAGTGCTATCAT
COX2	CACCCTGACATAGACAGTGAAAG	CTGGGTCACGTTGGATGAGG

### Immunofluorescence (IF) staining

For BV-2 microglial cells, sample preparation involved fixation, permeabilization, and blocking to ensure specificity. Primary antibodies targeting antigen were incubated with cells overnight followed by the addition of secondary antibodies conjugated with fluorescent dyes. The excess antibodies were washed away and nuclear staining was performed with diaminidine phenyl indole (DAPI). Prepared cells were mounted on slides, then examined with a fluorescence microscope (Leica, Oskar, Germany) and images captured to reveal the distribution and localization of target antigen within the cells. For spinal cord tissues, paraffin section underwent deparaffinization and rehydration, followed by blocking with buffer containing bovine serum albumin (BSA) for 1 h. The primary antibodies specific for target antigens were applied and incubated at 4 °C overnight. The next day, secondary antibodies, labeled with a fluorescent dye, were applied and cell nuclei stained with DAPI. The prepared sections were mounted and examined with a fluorescence microscope (Leica) to capture images. Regarding the quantification of fluorescence intensity, the brief method is as follows: Import the immunofluorescence image into ImageJ and then apply thresholding to distinguish fluorescent signal from background noise. Use the selection tools in ImageJ to outline ROIs within the image, such as individual cells or tissue structures. Once ROIs are defined, use ImageJ’s measurement tools to quantify fluorescence intensity or other relevant parameters within the selected regions and finally compare fluorescence levels between experimental groups or conditions.

### Intracellular ROS detection

After adding HMGB1 to CAPE-pretreated BV-2 microglial cells, a 10 μM DCFH-DA probe (Beyotime, Nanjing, China) was added to the cells for 30 min and intracellular ROS concentration assessed using a fluorescence microscope (Leica).

### Detection of TNF-a and IL-1β

Utilize the ELISA assay kit (YiFeiXue Biotechnology, Nanjing, China) to measure the levels of TNF-α and IL-1β in spinal cord tissue according to the manufacturer's instructions.

### Mitochondrial membrane potential measurement

A JC-1 kit (ab113850, Abcam, Cambridge, MA, USA) was used to detect the mitochondrial membrane potential of BV-2 microglial cells. CAPE-pretreated cells were seeded in a 24-well plate and then stained with JC-1 by incubation in the working solution for 20 min. A fluorescence microscope (Leica) was used to measure the fluorescence intensity of each group of cells. JC-1 forms aggregates with red fluorescence (in healthy mitochondria). With decreased membrane potential, JC-1 became a monomer and exhibits green fluorescence. Change in the ratio of red to green fluorescence was used as an indicator of mitochondrial condition.

### Molecular docking

The PubChem (https://pubchem.ncbi.nlm.nih.gov/) and RCSB databases (https://www.rcsb.org/) were used to forecast the downstream proteins of CAPE (PubChem CID: 5,281,787). Molecular docking was performed by ligand docking in the Glide module in Schrodinger Maestro software.

### Histopathology

For hematoxylin and eosin (HE) staining, deparaffinization was performed and then the slides were immersed in hematoxylin, which stains cell nuclei blue-purple. After differentiation and bluing, slides were subjected to eosin staining to color cytoplasm and extracellular structures in shades of pink and red. Following dehydration and clearing with xylene, the slides were mounted with coverslips. For Nissl staining, the cord tissue sections were mounted on slides, deparaffinized, and then submerged in a Cresyl Violet solution to specifically stain Nissl bodies. After rinsing and dehydration, sections were cleared with an agent like xylene and mounted with coverslips. The stained tissue sections were observed with a light microscope (Leica) and the dark blue neuron Nissl bodies were visible. For Luxol Fast Blue (LFB) staining, the cord tissue underwent deparaffinization and rehydration before immersion in LFB solution. After rinsing, the sections were dehydrated, cleared, mounted with coverslips, and observed with a light microscope (Leica). Quantification of tissue was analyzed by ImageJ software. In brief, import the image into ImageJ and apply thresholding to distinguish stained regions from the background. Use the selection tools in ImageJ to outline ROIs within the image such as tissue structures. Once ROIs are defined, use ImageJ's measurement tools to quantify staining intensity or other relevant parameters within the selected regions.

### Behavioral assessment

Motor function after SCI was evaluated based on the Basso Mouse Scale (BMS) [26] and footprint analysis. BMS scores were assessed at 1, 3, 7, 14, 21, and 28 days after SCI (dpi) to evaluate motor recovery for the different groups. Footprint analysis was performed at 28 dpi, which evaluated dragging of hind limbs, stride length, and stride width as functions of motor recovery in mice treated or not with CAPE.

### Statistical analysis

All statistical analysis was performed using GraphPad Prism 8.3. The means ± standard deviations of the data are displayed, and all experiments were repeated in at least three separate experiments. Comparisons between two groups were analyzed by *t*-tests and among more than two groups using one-way ANOVAs followed by Tukey’s post hoc test. Differences were considered significant when *p < 0.05. *p < 0.05, **p < 0.01, ***p < 0.001, ****p < 0.0001 and n.s. = no significance.

## Results

### CAPE reduced mouse pathology and improved motor function after SCI

**Fig. 1:**
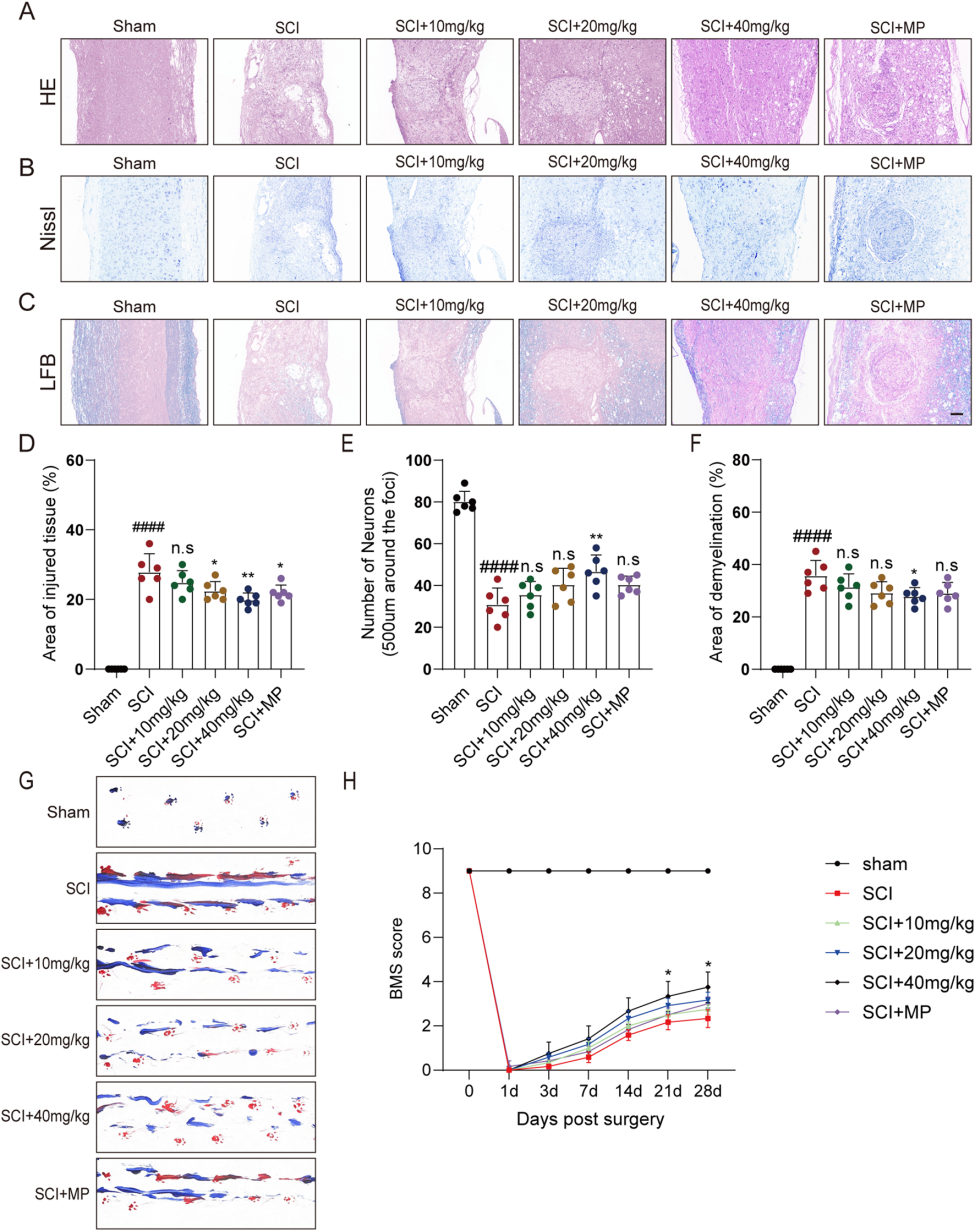
CAPE reduced mouse pathology and improved motor function after SCI. **A** HE staining images of the spinal cord centered around the injured core (3 mm) obtained at 28 dpi; Scale bar = 200, 100 μm. **B** Representative images of Nissl stained sections obtained from longitudinal sections centered at the injured core (3 mm) at 28 dpi. Scale bar = 200, 100 μm. **C** Representative images of LFB stained sections obtained from longitudinal sections centered around the injured core (3 mm) at 28 dpi; Scale bar = 200, 100 μm. **D** Quantitative analysis of the lesion area at 28 dpi; n = 6. **E** Quantitative analysis of survived neurons at 28 dpi; n = 6. **F** Quantitative analysis of the demyelinated area at 28 dpi; n = 6. **G** Footprint images of Sham, SCI, CAPE-treated and MP-treated SCI mice performed at 28 dpi. **H** BMS scores within 28 dpi in Sham, SCI, CAPE-treated and MP-treated SCI mice. Data are shown as means ± SEM. Statistical significance was determined with one-way ANOVA followed by Tukey’s post hoc test. ^**#**^p < 0.05 vs. Sham group, *p < 0.05 vs. SCI group, *p < 0.05, **p < 0.01, ***p < 0.001, ****p < 0.0001, n.s. = no significance

In order to visually assess the effect of CAPE on neural structures after SCI, tissues were evaluated by pathologic staining methods including HE, Nissl, and LFB at 28 dpi and MP was used as a positive control. Surprisingly, HE staining showed that treatment with CAPE significantly reduced the area of nervous tissue defects, the therapeutic effect was similar to that of MP. The reduction in nervous tissue defects was enhanced at the greater dose of CAPE (Fig. 1A, D). Although SCI caused the death of most neurons within and around the damaged area, treatment with 40 mg/kg CAPE rescued more surviving neurons (Fig. 1B, E). LFB staining demonstrated CAPE to inhibit demyelination, especially at 40 mg/kg (Fig. 1C, F). Footprint analysis and BMS scores were evaluated to assess the therapeutic effect of CAPE on motor functional recovery of SCI mice. Footprint diagrams showed stride length and stride width of SCI mice treated with 10, 20 or 40 mg/kg CAPE to be improved and the dragging of the hind limbs was mitigated compared to the injured group (Fig. 1G). At 21 and 28 days after injury, mice treated with 40 mg/kg CAPE displayed higher BMS scores than those without CAPE (Fig. 1H).

### CAPE inhibited SCI-induced microglial neuro-inflammation and oxidative stress

**Fig. 2:**
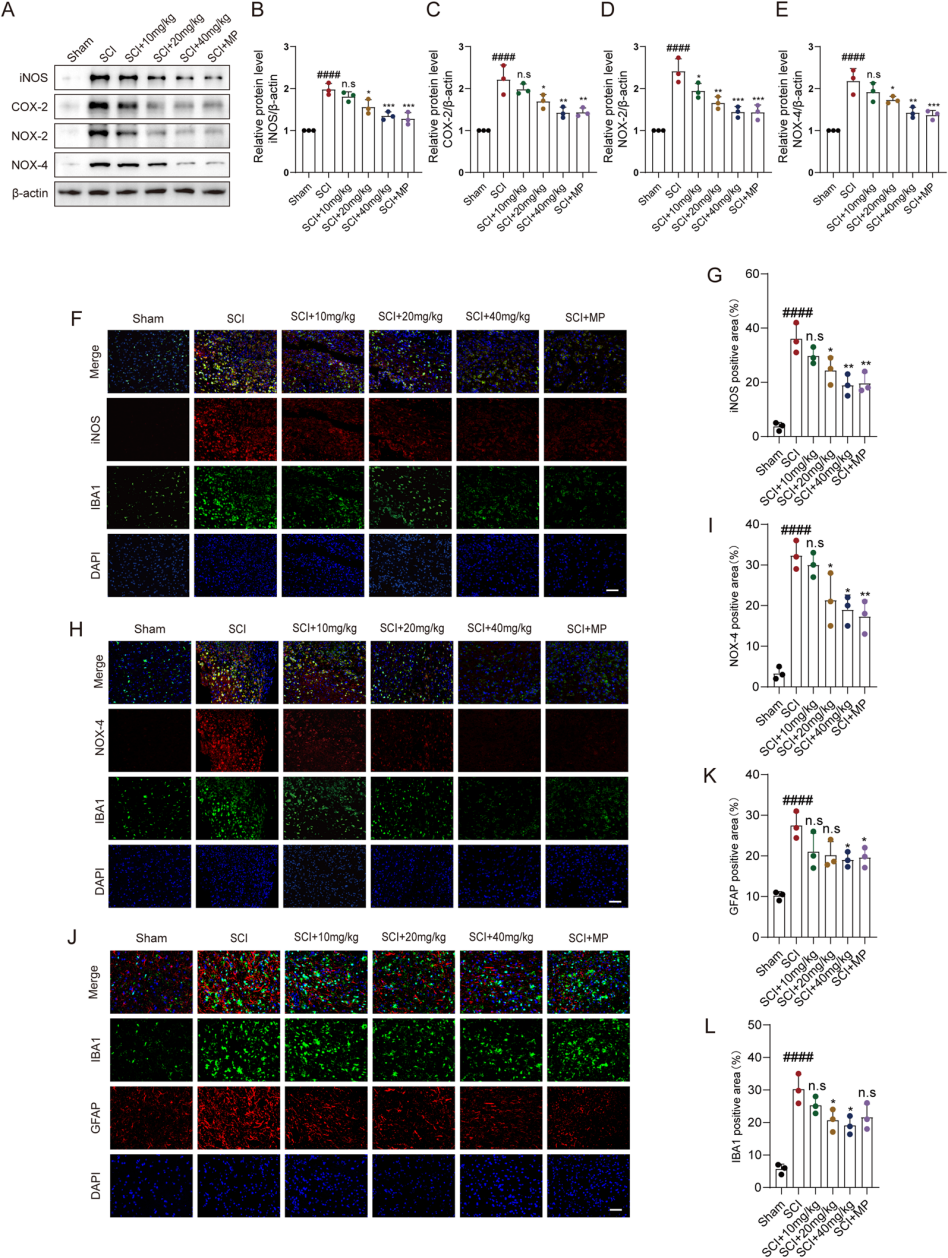
CAPE inhibited SCI-induced microglial neuro-inflammation and oxidative stress. **A** WB analysis of iNOS, COX-2, NOX-2, and NOX-4 levels in injured spinal cords at 7 dpi; n = 3. β-actin was used as the control. **B** Bar graph showing a quantitative analysis of iNOS expression; n = 3. **C** Bar graph showing a quantitative analysis of COX-2 expression; n = 3. **D** Bar graph showing a quantitative analysis of NOX-2 expression; n = 3. **E** Bar graph showing a quantitative analysis of NOX-4 expression; n = 3. **F** Double immunofluorescence labeling of microglia for IBA-1(green) and iNOS (red), obtained from longitudinal Sects. 1 mm caudal to the lesion site at 7 dpi. Scale bar = 50 μm. **G** Quantitative analysis of iNOS fluorescence intensity at 7 dpi.** H**, Double immunofluorescence labeling of microglia for IBA-1(green) and NOX-4 (red), obtained from longitudinal Sects. 1 mm caudal to the lesion site at 7 dpi. Scale bar = 50 μm. **I** Quantitative analysis of NOX-4 fluorescence intensity at 7 dpi. **J** Double immunofluorescence labeling of microglia for IBA-1 (red) and astrocytes for GFAP (green) obtained from longitudinal sections centered around the injured core (3 mm) at 7 dpi; Scale bar = 50 μm. **K** Quantitative analysis of the area of astrocyte scar at 7 dpi. **L** Quantitative analysis of the area of microglia scar at 7 dpi. Data are shown as means ± SEM. Statistical significance was determined with one-way ANOVA followed by Tukey’s post hoc test. ^**#**^p < 0.05 vs. Sham group, *p < 0.05 vs. SCI group, *p < 0.05, **p < 0.01, ***p < 0.001, ****p < 0.0001, n.s. = no significance

After SCI, activated microglia release iNOS and COX-2. These aggravate the neuro-inflammatory response and induce oxidative stress. WB showed that iNOS and COX-2 protein levels were increased post-SCI. As with the anti-inflammatory and antioxidant effects of MP, CAPE treatment effectively decreased SCI-induced iNOS and COX-2, which indicates that CAPE significantly inhibited neuro-inflammation after SCI. With 40 mg/kg CAPE treatment, the inhibition was more obvious (Fig. 2A–C). Compared to the injury group, CAPE significantly reduced levels of oxidative stress-related proteins, NOX-2 and NOX-4, after SCI (Fig. 2A, D, E). ELISA assay further showed that the levels of inflammatory cytokines TNF-α and IL-1β in the injured spinal cord were significantly reduced after CAPE treatment (Additional file 1: Fig. S1). By IF staining we explored whether CAPE inhibited microglia-mediated neuro-inflammation and oxidative stress. The microglia biomarker, IBA-1, was co-stained with iNOS and NOX-4. IF showed that CAPE decreased the fluorescence intensity of iNOS co-stained with IBA-1, compared to the injury group, with the effects more dramatic at the greater does of CAPE (Fig. 2F, G). By IF, CAPE rescued the expression of NOX-4 in microglia induced by SCI (Fig. 2H, I). The severity of neuro-inflammation after SCI was also reflected in the formation of glial scars. IF results demonstrated both IBA-1^+^ and GFAP^+^ areas to be significantly decreased by 40 mg/kg CAPE treatment (Fig. 2J–L). Apoptosis caused by inflammation and oxidative stress hinders nerve repair after SCI so we further examined the expression levels of apoptosis-related proteins Bax and Bcl-2 after SCI. The WB results showed that 40 mg/kg CAPE treatment could significantly reduce the expression of pro-apoptotic protein Bax and increase the expression of anti-apoptotic protein Bcl-2, indicating that CAPE could effectively regulate the apoptosis process after SCI (Additional file 2: Fig. S2).

### CAPE suppressed neuro-inflammation and oxidative stress of HMGB1-stimulated BV-2 microglia in vitro

**Fig. 3:**
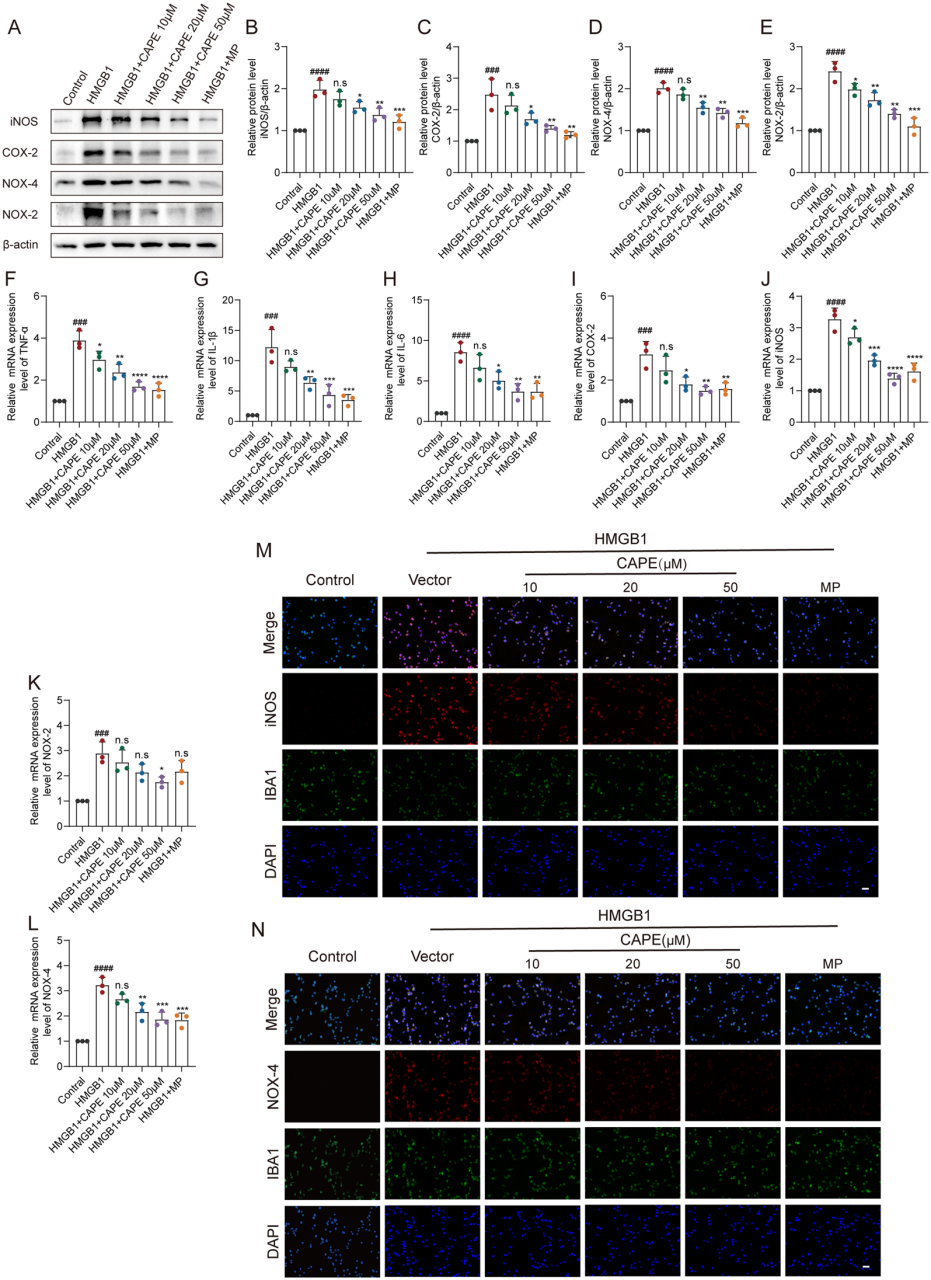
CAPE suppressed neuro-inflammation and oxidative stress of HMGB1-stimulated BV-2 microglia in vitro. **A** WB analysis of iNOS, COX-2, NOX-2, and NOX-4 levels in HMGB1-stimulated BV-2 microglial cells after CAPE or MP treatment for 3 h; n = 3. β-actin was used as the control. **B** Bar graph showing a quantitative analysis of iNOS expression; n = 3. **C** Bar graph showing a quantitative analysis of COX-2 expression; n = 3. **D** Bar graph showing a quantitative analysis of NOX-4 expression; n = 3. **E** Bar graph showing a quantitative analysis of NOX-2 expression; n = 3. **F**–**H** Relative mRNA levels of TNF-α, IL-1β, and IL-6 in HMGB1-stimulated BV-2 microglial cells after CAPE or MP treatment for 3 h; n = 3. **I**–**L** Relative mRNA levels of PTGS2, NOS2, NOX-2, and NOX-4, respectively, in HMGB1-stimulated BV-2 microglial cells after CAPE or MP treatment for 3 h; n = 3. **M** Representative immunofluorescence images of iNOS (red) and IBA-1 (green) in HMGB1-stimulated BV-2 microglial cells after CAPE or MP treatment for 3 h; n = 3. **N** Representative immunofluorescence images of NOX-4 (red) and IBA-1 (green) in HMGB1-stimulated BV-2 microglial cells after CAPE or MP treatment for 3 h; n = 3. Data are shown as means ± SEM. Statistical significance was determined with one-way ANOVA followed by Tukey’s post hoc test. ^**#**^p < 0.05 vs. Control group, *p < 0.05 vs. HMGB1 group, *p < 0.05, **p < 0.01, ***p < 0.001, ****p < 0.0001, n.s. = no significance

HMGB1 was used to activate BV-2 microglia in vitro to simulate the neuro-inflammatory response that follows SCI. WB results demonstrated HMGB1 to markedly increase protein levels of iNOS, COX-2, NOX-2, and NOX-4, which indicates that HMGB1 successfully induced microglial neuro-inflammation and oxidative stress in vitro. In contrast, CAPE administration significantly reduced the levels of these mediators in HMGB1-stimulated BV-2 microglia, similar results were obtained with MP treatment. (Fig. 3A–E). Levels of TNF-α, IL-1β, and 1L-6 mRNA, major components of the microglial inflammatory storm, were also evaluated by qPCR. Results showed that CAPE markedly inhibited the mRNA levels of these inflammatory cytokines (Fig. 3F–H).In addition, CAPE reduced mRNA expression levels of COX-2, iNOS, NOX-2, and NOX-4, with results similar to WB results (Fig. 3I–L). Further, IF staining demonstrated CAPE to reduce HMGB1-induced iNOS and NOX-4 levels (Fig. 3M, N). These results demonstrate the inhibitory effect of CAPE on microglial neuro-inflammation and oxidative stress.

### CAPE reduced mitochondrial damage by regulation of the SIRT1/PGC1α/DRP1 axis

**Fig. 4:**
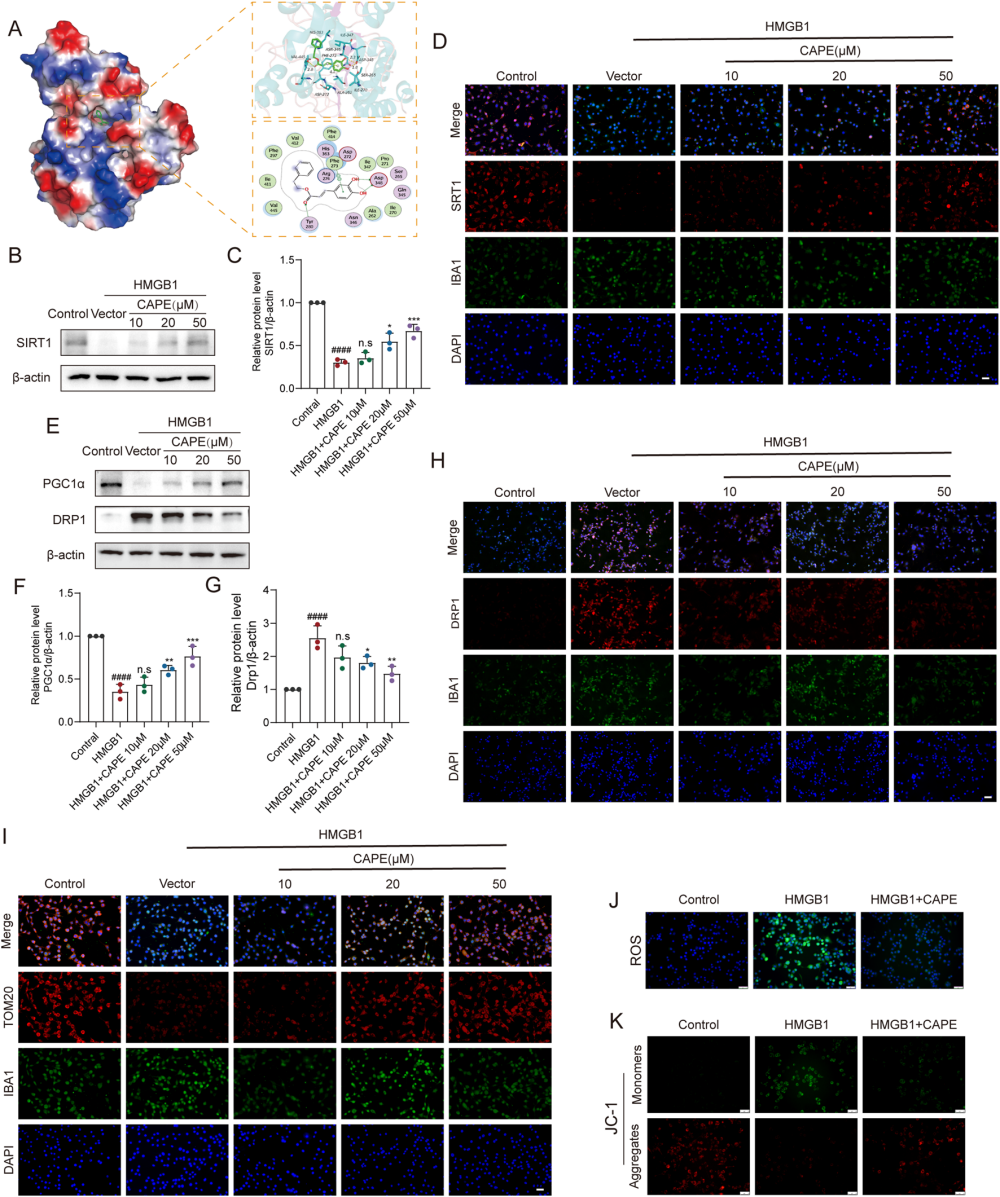
CAPE reduced mitochondrial damage by regulation of the SIRT1/PGC1α/DRP1 axis. **A** Binding of SIRT1 and CAPE within the 3D structure of the complex, the electrostatic surface of proteins and the detail binding mode of ligand with protein. **B** WB analysis of SIRT1 levels in HMGB1-stimulated BV-2 microglial cells after CAPE treatment for 3 h; n = 3. β-actin was used as the control. **C** Bar graph showing a quantitative analysis of SIRT1 expression; n = 3. **D** Representative immunofluorescence images of SIRT1 (red) and IBA-1 (green) within HMGB1-stimulated BV-2 microglial cells after CAPE treatment for 3 h; n = 3. **E** WB analysis of DRP1 and PGC1α levels within HMGB1-stimulated BV-2 microglial cells after CAPE treatment for 3 h; n = 3. β-actin was used as the control.** F**, Bar graph showing a quantitative analysis of PGC1α expression; n = 3. **G** Bar graph showing a quantitative analysis of DRP1 expression; n = 3. **H** Representative immunofluorescence images of DRP1 (red) and IBA-1 (green) within HMGB1-stimulated BV-2 microglial cells after CAPE treatment for 3 h; n = 3. **I** Representative immunofluorescence images of TOM20 (red) and IBA-1 (green) within HMGB1-stimulated BV-2 microglial cells after CAPE treatment; n = 3. Scale bar = 50 μm. **J** Representative immunofluorescence images of ROS (green) within HMGB1-stimulated BV-2 microglial cells after CAPE treatment; Scale bar = 50 μm. **K** Microglial mitochondrial membrane potential analyzed by JC-1 staining; Scale bar = 50 μm. Data are shown as means ± SEM. Statistical significance was determined with one-way ANOVA followed by Tukey’s post hoc test. ^**#**^p < 0.05 vs. Control group, *p < 0.05 vs. HMGB1 group, *p < 0.05, **p < 0.01, ***p < 0.001, ****p < 0.0001, n.s. = no significance

The specific molecular mechanisms by which CAPE reduced cell and tissue damage was investigated. Studies have shown that CAPE ameliorates myocardial ischemia/reperfusion injury by suppressing oxidative stress and the inflammatory response through the SIRT1/eNOS/NF-κB pathway [27]. Further, CAPE has been shown to play a neuroprotective role by activation of SIRT1 expression in peripheral sensory neuropathy [28]. To assess the interaction of CAPE and SIRT1, we used Schrodinger Maestro to simulate molecular docking of CAPE and SIRT1. CAPE bound to the active pocket of SIRT1 (binding energy: − 7.31 kcal/mol) (Fig. 4A). WB results showed that HMGB1 reduced protein levels of SIRT1, with reversal by 20 and 50 μM CAPE. Further, CAPE significantly promoted activation of SIRT1 (Fig. 4B, C). These results were confirmed by IF staining (Fig. 4D). Previous studies have shown that the activation of SIRT1 induces activation of PGC1α, which plays an anti-inflammatory and anti-oxidant role in several diseases [29]. In this study, WB results demonstrated CAPE to promote protein levels of PGC1α while activating SRIT1 (Fig. 4E, F). PGC1α has been shown to inhibit phosphorylation of DRP1, which is a key protein in the regulation of mitochondrial division. WB results showed that HMGB1 increased DRP1 protein levels in microglia, suggesting mitochondrial division, while CAPE inhibited this process (Fig. 4E, G). IF staining of DRP1 produced similar results (Fig. 4H). The mitochondrial protein, TOM20, maintains mitochondrial biogenesis and normal function. IF results showed that CAPE treatment rescued the reduced levels of TOM20 induced by HMGB1 (Fig. 4I). We next examined whether CAPE inhibited the mitochondria damage associated with inflammatory microglia. Intracellular ROS levels were significantly elevated after HMGB1 stimulation, with CAPE treatment significantly inhibiting the level of ROS (Fig. 4J). The JC-1 probe was used to evaluate mitochondrial membrane potential and in mice treated with CAPE, higher mitochondrial membrane potential was found compared to untreated mice (Fig. 4K). These results indicate that CAPE reduces mitochondrial damage by activation of SIRT1 and PGC1α and by inhibition of DRP1 phosphorylation within inflammatory microglia.

### After mouse SCI, CAPE effectively regulated the SIRT1/PGC1α/DRP1 signaling axis

**Fig. 5:**
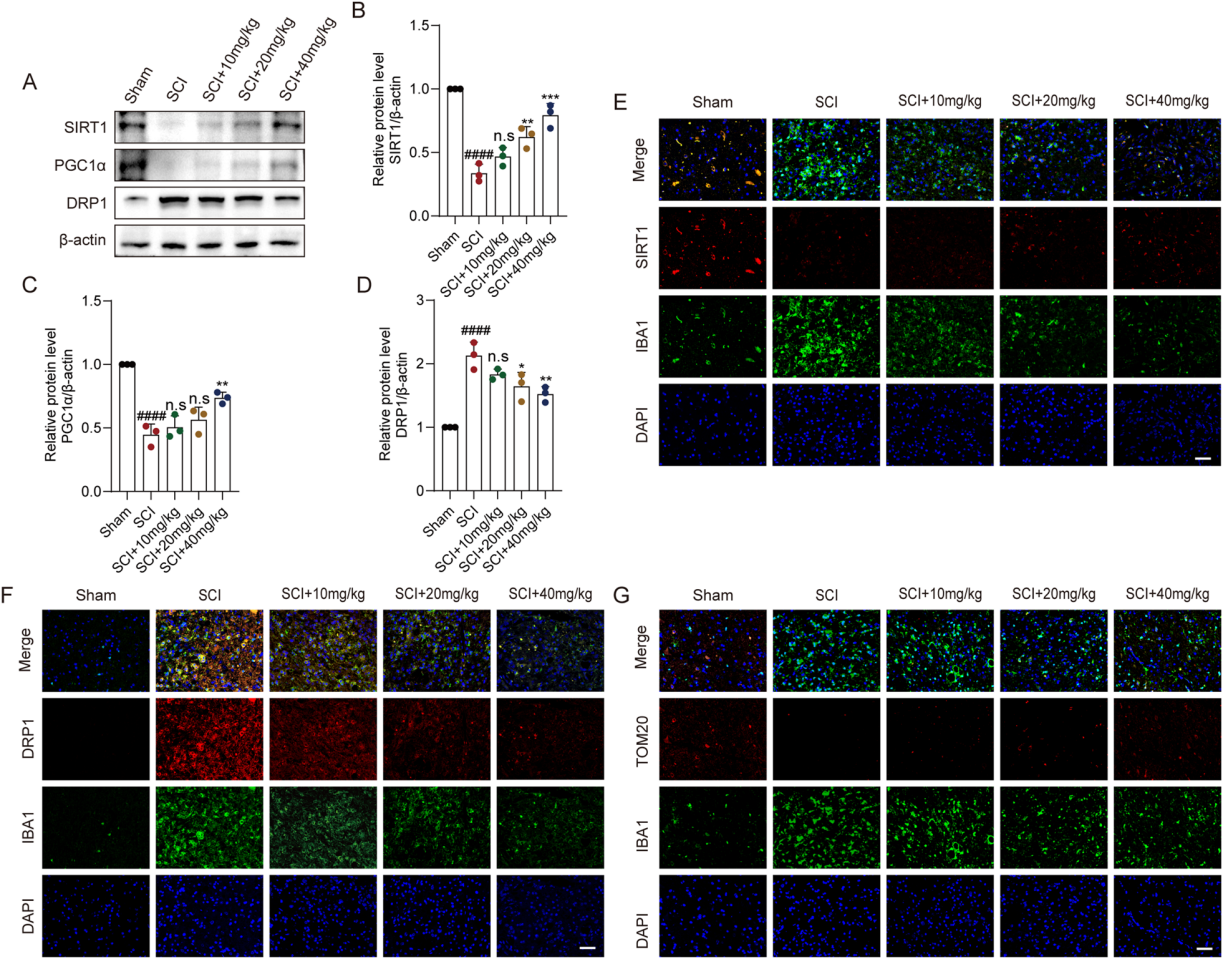
After mouse SCI, CAPE effectively regulated the SIRT1/PGC1α/DRP1 signaling axis. **A** WB analysis of SIRT1, PGC1α, and DRP1 levels in injured spinal cords at 7 dpi; n = 3. β-actin was used as the control. **B** Bar graph showing a quantitative analysis of SIRT1 expression; n = 3. **C** Bar graph showing a quantitative analysis of PGC1α expression; n = 3. **D** Bar graph showing a quantitative analysis of DRP1 expression; n = 3. **E** Double immunofluorescence labeling of microglia for IBA-1(green) and SIRT1 (red), obtained from longitudinal Sects. 1 mm caudal to the lesion site at 7 dpi. Scale bar = 50 μm. **F** Double immunofluorescence labeling of microglia for IBA-1(green) and DRP1 (red), obtained from longitudinal Sects. 1 mm caudal to the lesion site at 7 dpi. Scale bar = 50 μm. **G** Double immunofluorescence labeling of microglia for IBA-1(green) and TOM20 (red), obtained from longitudinal Sects. 1 mm caudal to the lesion site at 7 dpi. Scale bar = 50 μm. Data are shown as means ± SEM. Statistical significance was determined with one-way ANOVA followed by Tukey’s post hoc test. ^**#**^p < 0.05 vs. Sham group, *p < 0.05 vs. SCI group, *p < 0.05, **p < 0.01, ***p < 0.001, ****p < 0.0001, n.s. = no significance

To further support the findings of the in vitro experiments, we assessed the SIRT1/PGC1α/DRP1 signaling axis in vivo. By WB analysis, a significant decrease in levels of SIRT1 and PGC1α and a notable increase in DRP1 levels were observed after SCI. With 40 mg/kg CAPE treatment, SIRT1 and PGC1α levels increased and DRP1 levels decreased (Fig. 5A–D). Within injured spinal cord tissues, IF staining demonstrated CAPE treatment to increase SIRT1 levels and decrease DRP1 levels in microglia (Fig. 5E, F). Further, although TOM20 levels were significantly lower after SCI compared to the Sham group, TOM20 levels were increased after CAPE treatment, indicating that CAPE successfully rescued damaged mitochondrial structure (Fig. 5G). These findings demonstrate CAPE to modulate the expression of the SIRT1/PGC1α/DRP1 signaling axis after SCI, which may be the primary molecular mechanism underlying CAPE's SCI therapeutic effects.

### The effects of CAPE after SCI were dependent on SIRT1

**Fig. 6:**
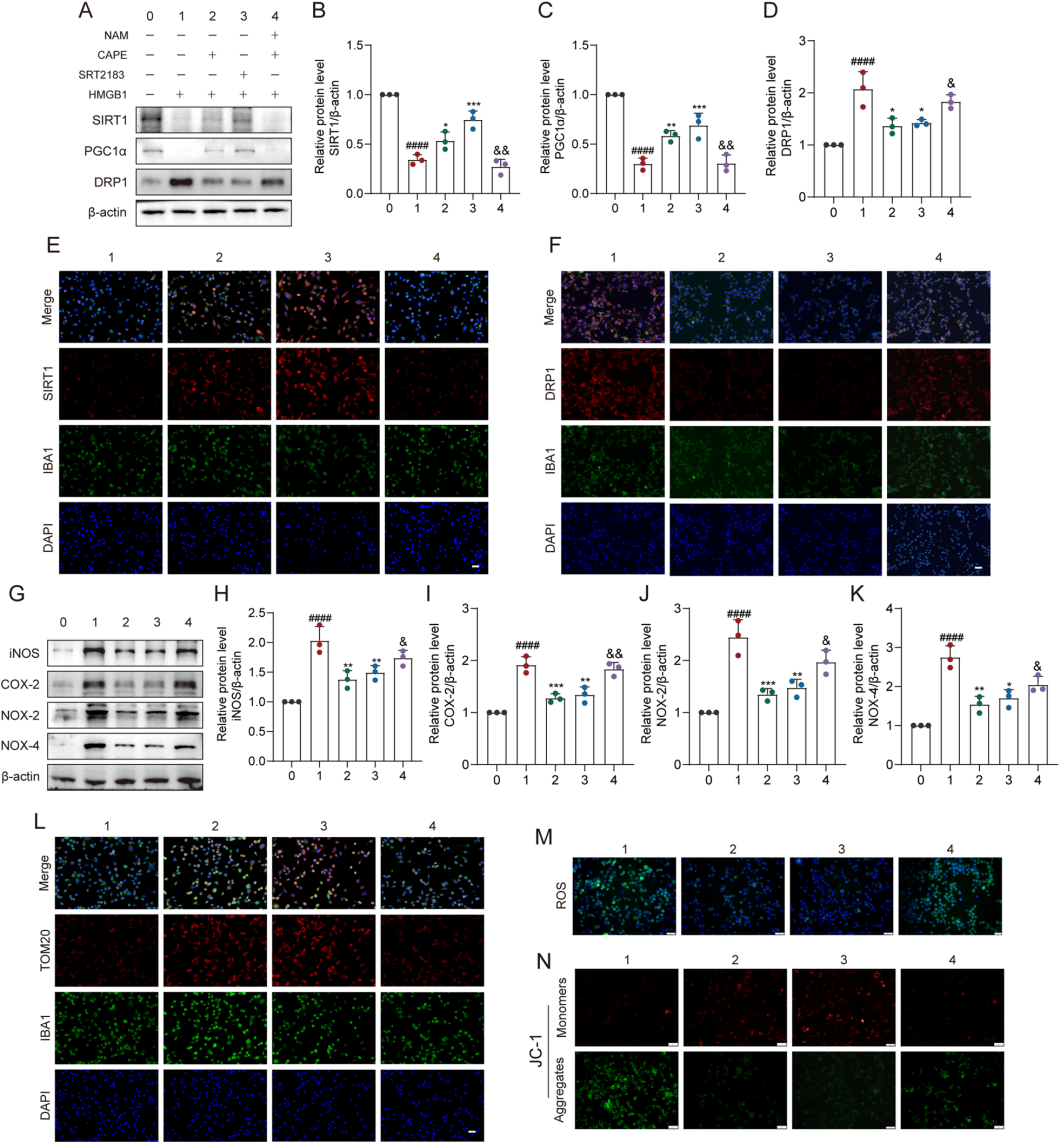
The effects of CAPE after SCI were dependent on SIRT1. **A** WB analysis of SIRT1, PGC1α, and DRP1 levels in HMGB1-stimulated BV-2 microglial cells after CAPE, NAM and SRT2183 treatment; n = 3. β-actin was used as the control. **B** Bar graph showing a quantitative analysis of SIRT1 expression; n = 3. **C** Bar graph showing a quantitative analysis of PGC1α expression; n = 3. **D** Bar graph showing a quantitative analysis of DRP1 expression; n = 3. **E** Representative immunofluorescence labeling images for SIRT1 (red) and IBA-1 (green) within HMGB1-stimulated BV-2 microglial cells after CAPE, NAM and SRT2183 treatment; n = 3. Scale bar = 50 μm. **F** Representative immunofluorescence labeling images for DRP1 (red) and IBA-1 (green) within HMGB1-stimulated BV-2 microglial cells after CAPE, NAM and SRT2183 treatment; n = 3. Scale bar = 50 μm. **G** WB analysis of iNOS, COX-2, NOX-2, and NOX-4 levels in HMGB1-stimulated BV-2 microglial cells after CAPE, NAM and SRT2183 treatment; n = 3. β-actin was used as the control. **H** Bar graph showing a quantitative analysis of iNOS expression; n = 3. **I** Bar graph showing a quantitative analysis of COX-2 expression; n = 3. **J** Bar graph showing a quantitative analysis of NOX-2 expression; n = 3. **K** Bar graph showing a quantitative analysis of NOX-4 expression; n = 3. **L** Representative immunofluorescence labeling images of TOM20 (red) and IBA-1 (green) within HMGB1-stimulated BV-2 microglial cells after CAPE, NAM and SRT2183 treatment; n = 3. Scale bar = 50 μm. **M** Representative immunofluorescence labeling images of ROS (green) within HMGB1-stimulated BV-2 microglial cells after CAPE, NAM and SRT2183 treatment; Scale bar = 50 μm. **N** Microglial mitochondrial membrane potential analyzed by JC-1 staining; Scale bar = 50 μm. Data are shown as means ± SEM. Statistical significance was determined with one-way ANOVA followed by Tukey’s post hoc test. ^**#**^p < 0.05 vs. Control group, *p < 0.05 vs. HMGB1 group, ^&^p < 0.05 vs. CAPE group, *p < 0.05, **p < 0.01, ***p < 0.001, ****p < 0.0001, n.s. = no significance

To further demonstrate the inhibitory effect of CAPE on inflammatory microglia via SIRT1, we used the SIRT1 inhibitor nicotinamide (NAM) and SIRT1 agonist SRT2183. WB analysis demonstrated NAM to inhibit the protein levels of SIRT1 and PGC1α and to increase DRP1, while SRT2183 treatment obtained the opposite results.. The results indicate that NAM and SRT2183 can sufficiently regulate the SIRT1/PGC1α/DRP1 axis (Fig. 6A–D). Further, NAM significantly inhibited the fluorescence intensity of SIRT1 and reversed the CAPE-induced decrease in the fluorescence intensity of DRP1. These results indicate that CAPE did not alleviate mitochondrial damage when SIRT1 was inhibited (Fig. 6E, F). As demonstrated above, WB analysis showed that CAPE significantly inhibited iNOS, COX-2, NOX-2, and NOX-4 induced by HMGB1. However, when the SIRT1 inhibitor NAM was included, the levels of these inflammatory mediators and oxidative stress-related proteins were increased (Fig. 6G–K). IF results showed that NAM reduced CAPE treatment-induced ameliorative levels of TOM20 (Fig. 4L). Moreover, compared to the CAPE group, mitochondrial damage was aggravated in the NAM group, as indicated by increased intracellular ROS levels and decreased mitochondrial membrane potential (Fig. 6M, N). Taken together, these results suggest that CAPE exerts anti-inflammatory and anti-oxidative stress effects as well as alleviates mitochondrial damage by regulation of the SIRT1/PGC1α/DRP1 axis, and levels of SIRT1.

## Discussion

As a traumatic neurological disorder with a poor prognosis, SCI has long been a focus of research in neuroscience [30, 31]. Paralysis, sensory disturbance, and sexual dysfunction due to SCI have a devastating impact on patients and their families [32]. Hence, it is of paramount importance to develop a comprehensive understanding of the mechanisms underlying the onset and progression of SCI as well as the means by which to suitably treat patients. Secondary injury, including neuro-inflammation, oxidative stress, lipid peroxidation, and electrolyte imbalance are the primary contributors to the aggravated neurological damage and adverse neurological outcomes that follow SCI [4, 33]. Microglia-mediated neuro-inflammation and oxidative stress play a major adverse role in the acute phase of SCI [34, 35]. Microglia, as the guardians of the nervous system, are activated rapidly after SCI. Activated microglia release numerous inflammatory mediators and ROS, which aggravate the neuro-inflammatory response, resulting in the accumulation of ROS that causes mitochondrial damage and oxidative stress [36, 37]. Cells undergoing mitochondrial injury lose their normal physiological function. Further, toxic mediators are released by large numbers of dying cells, which further aggravate the neuro-inflammatory response [38, 39]. A vicious cycle of neuro-inflammation, oxidative stress, and mitochondrial damage make treatment of SCI very difficult. As such, intervention of microglia-mediated neuro-inflammation and oxidative stress after SCI is critical period to effective treatment of SCI patients.

At present, the clinical anti-inflammatory treatment of SCI is mainly limited to methylprednisolone pulse therapy [40, 41]. Although this treatment has a long history in clinical practice, there are adverse side effects such as hypertension, hyperglycemia, gastrointestinal bleeding, aggravation of infection, and symptoms of adrenocortical hyper-function [42, 43]. Moreover, studies have shown that high-dose methylprednisolone pulse therapy does not effectively improve the prognosis of SCI patients [44]. Therefore, identification of new anti-inflammatory drugs for SCI treatment is essential.

CAPE, one of the main bioactive components of propolis, has been reported to effectively repress tumor migration [45], relieve inflammatory responses [46], inhibit oxidation, and protect neurons from apoptosis [28, 47]. Although there have been many studies of the pharmacological effects of CAPE, there are few reports of its anti-inflammatory and anti-oxidative stress effects after SCI. Findings herein demonstrate CAPE to effectively suppress inflammatory mediator (iNOS, COX-2) and oxidative stress-related marker (NOX-2, NOX-4) production by activated microglia, both in vitro and in vivo. This suppression was accompanied by a reduction in inflammatory factors (TNF-α, IL-1β, IL-6). Furthermore, anti-inflammatory and anti-oxidative stress effects were enhanced with greater quantities of CAPE. These results suggest that CAPE inhibits microglia-driven neuro-inflammation and oxidative stress responses after SCI.

The SIRT1/PGC1α signaling pathway is a pivotal molecular cascade intricately involved in governing diverse cellular processes including energy metabolism, cellular oxidative stress responses, and inflammation [48–50]. At its core, SIRT1, a NAD^+^-dependent deacetylase belonging to the sirtuin family, exerts its influence by removing acetyl groups from specific target proteins, thereby regulating their activity [51, 52]. PGC1α is a transcriptional coactivator with the ability to modulate the expression of genes responsible for mitochondrial biogenesis, oxidative phosphorylation, and gluconeogenesis [53–55]. The functional synergy of the SIRT1/PGC1α pathway is evident in that SIRT1 activates PGC1α through deacetylation, leading to heightened PGC1α activity [56]. This synergy amplifies mitochondrial functionality, boosts energy production, and fortifies cells against oxidative stress and inflammation [57]. In this study, molecular docking analysis demonstrated a strong binding affinity between CAPE and SIRT1, suggesting that CAPE may regulate SIRT1 expression. In vitro and in vivo results demonstrated CAPE to enhance SIRT1 expression, concomitant with increased levels of PGC1α. Additionally, NAM, a SIRT1 inhibitor, was found to inhibit the regulatory effect of CAPE on SIRT1/PGC1α, indicating that the anti-inflammatory and anti-oxidative stress effects exerted by CAPE were dependent on SIRT1. PGC1α activation enhances mitochondrial biological function, and prior research has demonstrated PGC1α to suppress DRP1 expression [58], which is a critical cellular component responsible for regulation of mitochondrial fission, a process by which mitochondria divide into smaller organelles [59]. This study found that CAPE activated the SIRT1/PGC1α pathway and reduced the expression of DRP1, indicating that the process of mitochondrial fission induced by SCI is inhibited by CAPE. Further, CAPE substantially reduced intracellular and mitochondrial ROS levels while preserving mitochondrial membrane potential. These results suggest that CAPE safeguards normal mitochondrial function following SCI, potentially contributing to its anti-inflammatory and anti-oxidative stress effects. These observations warrant further investigation.

Many studies have shown that microglia-mediated neuro-inflammation and oxidative stress induce the release of neurotoxic substances, produce excessive glial scarring, and inhibit axon regeneration, which aggravate nerve tissue damage after SCI. This damage directly affects the recovery of motor function [60, 61]. In order to visually explore the therapeutic effect of CAPE after SCI, we continuously injected CAPE intraperitoneally into SCI mice and observed changes in nerve tissue structure and functional motor recovery. Results showed that CAPE treatment significantly reduced the area of nerve tissue defect and increased the number of surviving neurons, simultaneously mitigating the extent of demyelination, when compared to mice with injury alone. Notably, CAPE-treated mice exhibited improved motor function recovery and a more favorable prognosis following SCI, underscoring the potential therapeutic benefits of CAPE for SCI.

This study has shortcomings. First, although we confirmed associations between CAPE and SIRT1, the specific mechanism of action requires verification. Second, further experiments are necessary to validate CAPE's therapeutic potential for treating SCI. Finally, it is imperative to investigate whether CAPE influences other SCI related biological processes and to explore other regulatory mechanisms related to CAPE's impact on mitochondrial biogenesis.

## Conclusion

The current study demonstrated, for the first time, that CAPE alleviates microglia-mediated neuro-inflammation and oxidative stress, inhibits mitochondrial fission, protects mitochondrial biological function, and regulates the SIRT1/PGC1α/DRP1 signaling axis following SCI. In addition, CAPE was shown to effectively ameliorate nerve tissue damage and promote the recovery of motor function in SCI mice. This discovery offers a theoretical foundation for considering CAPE as a potential treatment for SCI, opening the door for future research into anti-inflammatory therapies for SCI.

### Supplementary Information

**Additional file 1: Figure S1.** The impact of CAPE on the expression of pro-inflammatory factors in SCI mice. **A** ELISA analysis of TNF-α level in injured spinal cords at 7 dpi; n = 3. **B** ELISA analysis of IL-1β level in injured spinal cords at 7 dpi; n = 3. Data are shown as means ± SEM. Statistical significance was determined with one-way ANOVA followed by Tukey’s post hoc test. #p < 0.05 vs. Sham group, *p < 0.05 vs. SCI group, *p < 0.05, **p < 0.01, ***p < 0.001, ****p < 0.0001, n.s. = no significance.

**Additional file 2:** **Figure S2.** CAPE inhibited the expression of apoptosis proteins after SCI. **A** WB analysis of Bax and Bcl-2 levels in injured spinal cords at 7 dpi; n = 3. β-actin was used as the control. **B** Bar graph showing a quantitative analysis of Bax expression; n = 3. **C** Bar graph showing a quantitative analysis of Bcl-2 expression; n = 3. Data are shown as means ± SEM. Statistical significance was determined with one-way ANOVA followed by Tukey’s post hoc test. #p < 0.05 vs. Sham group, *p < 0.05 vs. SCI group, *p < 0.05, **p < 0.01, ***p < 0.001, ****p < 0.0001, n.s. = no significance.

## Data Availability

The datasets used and/or analyzed during the current study are available from the corresponding author [Mingjie Xia], on reasonable request.
