# Supplementary figures and images for: Caffeic acid phenethyl ester inhibits neuro-inflammation and oxidative stress following spinal cord injury by mitigating mitochondrial dysfunction via the SIRT1/PGC1α/DRP1 signaling pathway
Source: J Transl Med. 2024 Mar 25;22:304. doi: 10.1186/s12967-024-05089-8 (PMC10962082; doi:10.1186/s12967-024-05089-8)

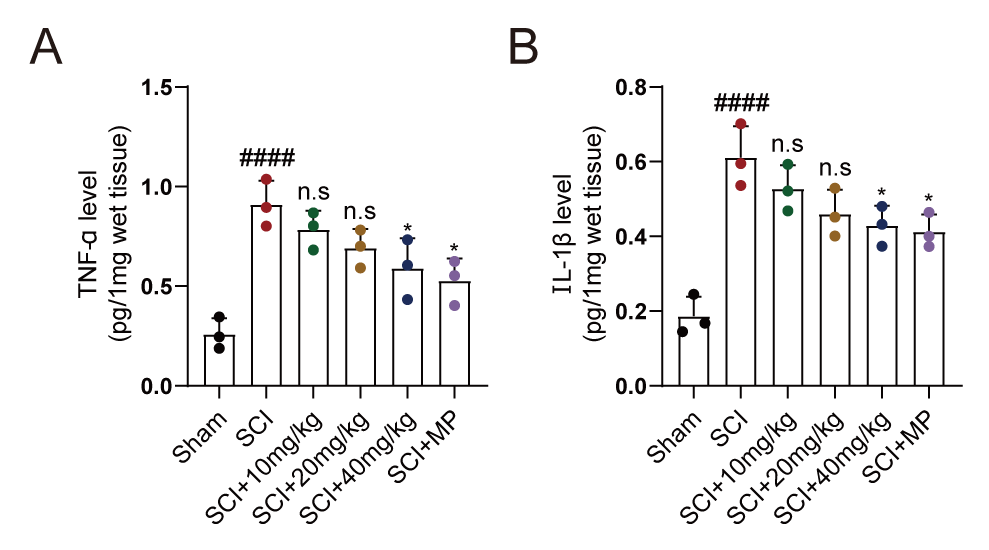

Supplement: Supplementary file 1 — Additional file 1: Figure S1. The impact of CAPE on the expression of pro-inflammatory factors in SCI mice. A ELISA analysis of TNF-α level in injured spinal cords at 7 dpi; n = 3. B ELISA analysis of IL-1β level in injured spinal cords at 7 dpi; n = 3. Data are shown as means ± SEM. Statistical significance was determined with one-way ANOVA followed by Tukey’s post hoc test. #p < 0.05 vs. Sham group, *p < 0.05 vs. SCI group, *p < 0.05, **p < 0.01, ***p < 0.001, ****p < 0.0001, n.s. = no significance. [file 12967_2024_5089_MOESM1_ESM.tif]

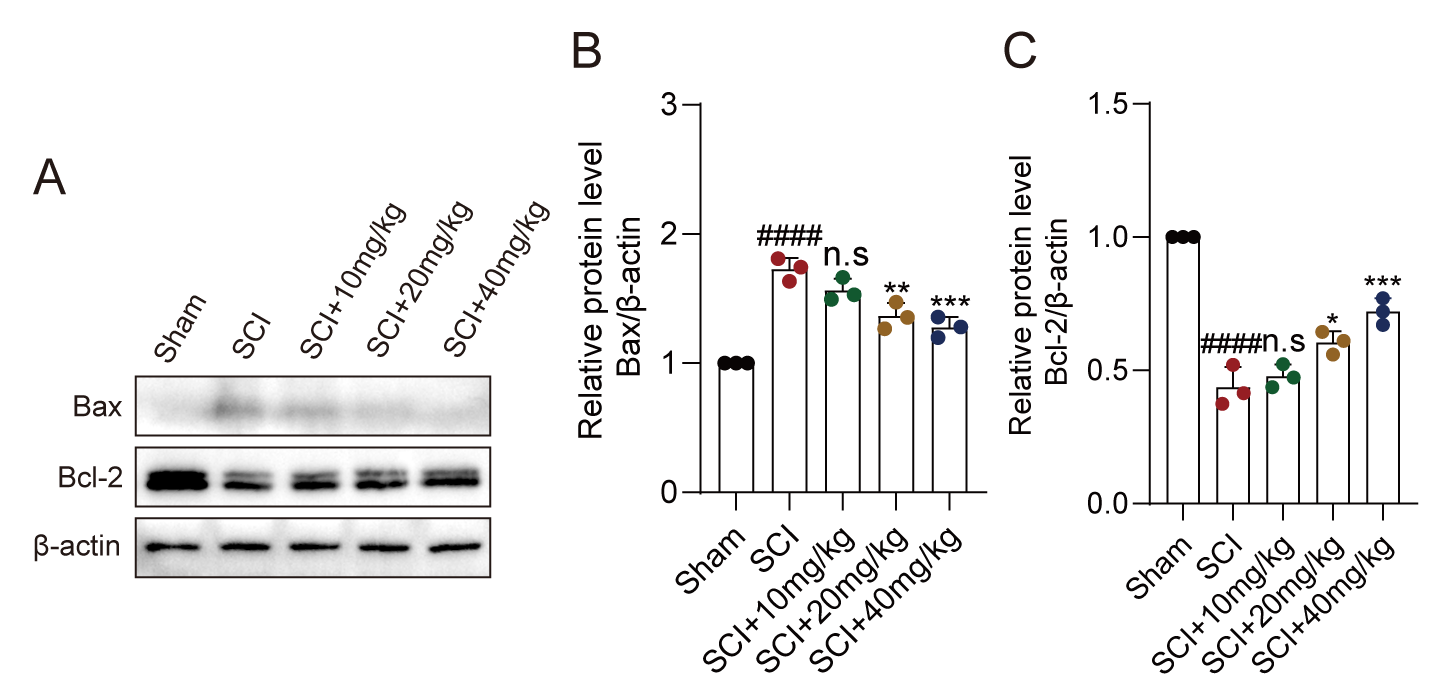

Supplement: Supplementary file 2 — Additional file 2: Figure S2. CAPE inhibited the expression of apoptosis proteins after SCI. A WB analysis of Bax and Bcl-2 levels in injured spinal cords at 7 dpi; n = 3. β-actin was used as the control. B Bar graph showing a quantitative analysis of Bax expression; n = 3. C Bar graph showing a quantitative analysis of Bcl-2 expression; n = 3. Data are shown as means ± SEM. Statistical significance was determined with one-way ANOVA followed by Tukey’s post hoc test. #p < 0.05 vs. Sham group, *p < 0.05 vs. SCI group, *p < 0.05, **p < 0.01, ***p < 0.001, ****p < 0.0001, n.s. = no significance. [file 12967_2024_5089_MOESM2_ESM.tif]
